# Treatment of ipsilateral high energy tibial plateau and calcaneal fractures by a circular wire fixator: a case report

**DOI:** 10.1186/1757-1626-2-7869

**Published:** 2009-06-03

**Authors:** Shabir Ahmed Dhar, Mohammed Farooq Butt, Murtaza Fazal Ali, Mohammed Ramzan Mir, Altaf Ahmed Kawoosa

**Affiliations:** Department of Orthopaedics, Government Medical CollegeSrinagar, Jammu and KashmirIndia

## Abstract

**Introduction:**

High energy tibial plateau fractures along with calcaneal fractures individually produce several challenges for the orthopaedic surgeon. The principles of bony reconstruction include anatomic reduction and rigid internal fixation of intra-articular fractures and accurate restoration of the coronal, sagittal and transverse mechanical axes. Due to the tenuous nature of the soft tissue and devitalisation of the comminuted fragments with open reduction, external fixation of type 6 tibial plateau fractures is recommended. We report a case with ipsilateral high energy tibial plateau and calcaneal fractures both of which were managed with an ilizarov ring fixator.

**Case presentation:**

A 55-year-old Kashmiri female presented to our department with an ipsilateral fracture of the tibial plateau and the calcaneum. Both were closed reduced and stabilized with an ilizarov ring fixator.

**Conclusion:**

The circular wire fixator provides a viable method to manage such fractures especially if they are co existent. This is especially true in situations where the soft tissue is compromised.

## Introduction

Fractures of the tibial plateau and the calcaneum sometimes occur together in the same patient because of the common causative axial load mechanism. Fractures of the calcaneus account for approximately 60% of tarsal injuries and usually are the result of a fall from height. It is necessary for other injuries of the appendicular skeleton be ruled out in all patients presenting with either a calcaneal or a tibial plateau fracture. The goal of the treatment in either fracture is the restoration of the articular congruity and axial alignment as also the achievement of joint stability and functional motion. This has to be done while allowing early range of motion and minimising wound complications. Closed manipulation restores the overall shape of the calcaneus, with emphasis on restoring the Bohler angle, obtaining facet congruency and re-establishing the normal heel width. This method is useful in patients who are not candidates for open treatment [1].

High energy tibial fractures are often associated with a high incidence of severe complications with traditional internal fixation [2].

We describe the management of a patient with high energy tibial and calcaneal fracture of the same limb, with an ilizarov fixator. The method provided manifest advantages in the management of the two high energy ipsilateral fractures sustained by our patient.

## Case presentation

A 55-year-old female presented to the out patient department of our hospital with a history of a fall from height. The patient had experienced immediate pain and swelling in the knee and ankle area of the limb. On removing the splint used to transport this patient and examining the patient, swelling of the knee, upper tibia and heel were noticed. Palpation produced crepitus of the upper tibia. Significant tenderness was elicited on palpation at the knee as well as the heel.

**Figure 1.**
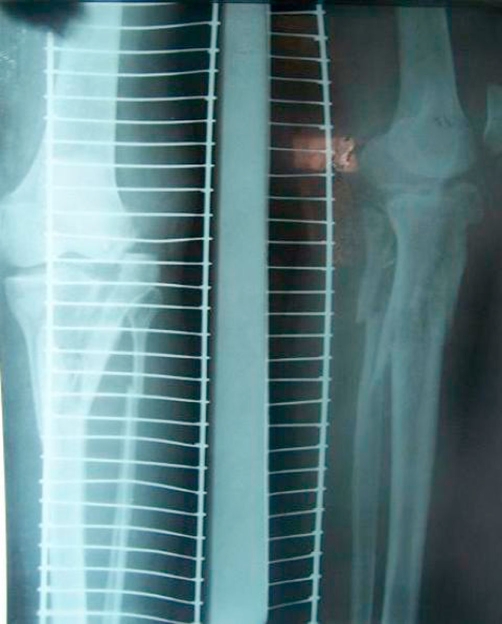
Showing the type 6 Schatzker fracture.

**Figure 2.**
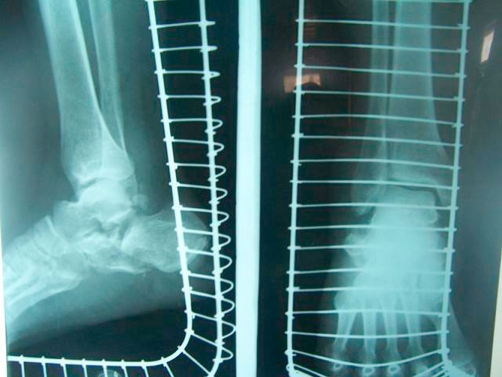
Showing the tongue type calcaneal fracture in the same patient.

Radiographs of the knee in the anterioposterior and lateral plane showed a type VI Schatzker fracture of the tibial plateau. Radiographic examination of the calcaneum showed a tongue type fracture of the calcaneum with complete loss of the Bohler angle (Figures 1 & 2).

The patient's limb was placed in a well padded splint and observed for compartment syndrome. Over a period of three days the patient developed significant ecchymosis around the upper tibia.

**Figure 3.**
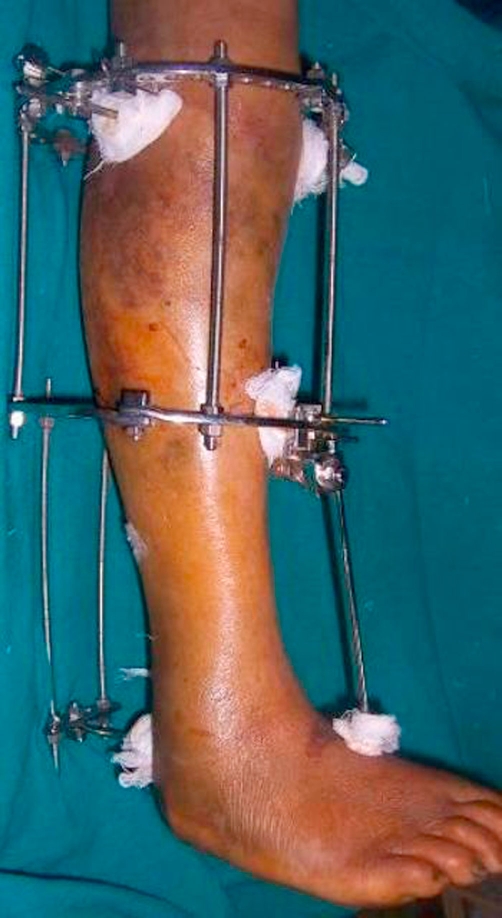
The Ilizarov ring fixator in place with the Schanz pin lifting the tongue fragment.

In view of the compromised soft tissue envelope, age of the patient and complexity of the trauma, it was decided to manage both the fractures in a ring fixator (Figure 3).

**Figure 4.**
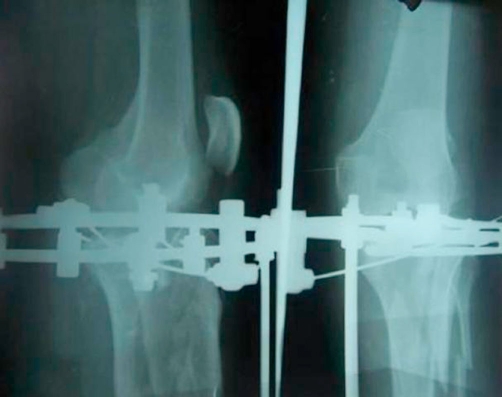
Radiograph of the tibial plateau as held by the hybrid fixation.

**Figure 5.**
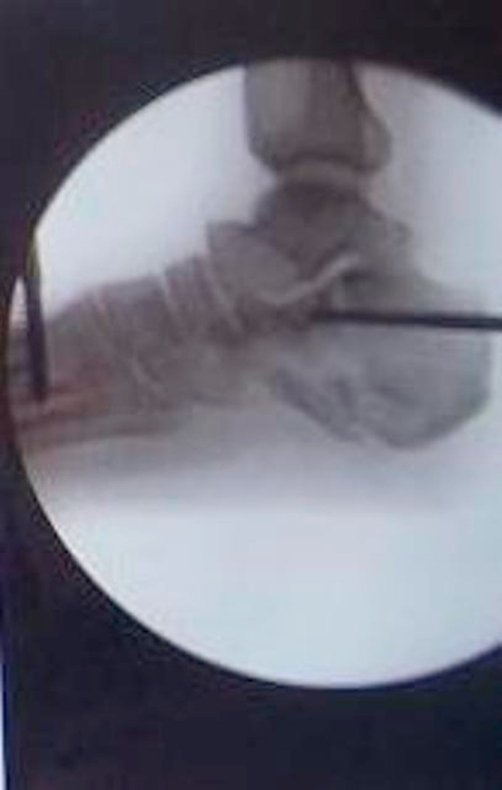
The calcaneal radiograph shows restoration of the Bohler angle.

The patient was anaesthetised and placed on a traction table. The proximal fragments were approximated with manual pressure under image intensifier control. Ilizarov wires were placed to obtain compression in the coronal plane. A cancellous transverse lag screw was placed to obtain further stability. An additional ring was placed and affixed to the bone, below the metaphysiodiaphysial comminution. This ring was affixed to the diaphyseal bone with two Schanz pins placed at right angles to each other. The foot was immobilised with a Schanz pin attached to this fixator. This Schanz pin was placed in the first metatarsal (Figures 4 & 5).

**Figure 6.**
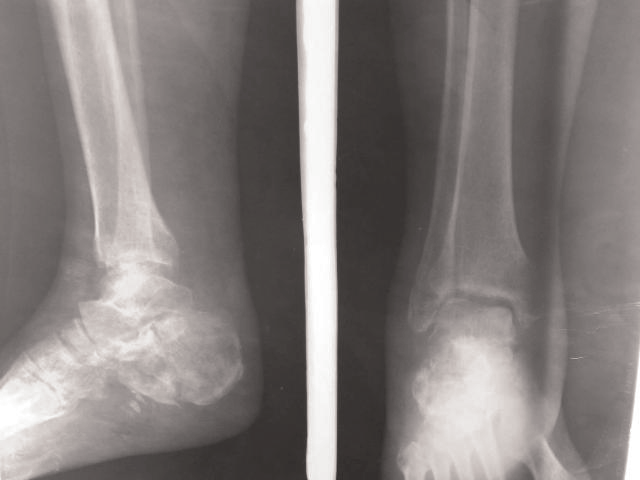
The calcaneus at the end of the treatment period.

**Figure 7.**
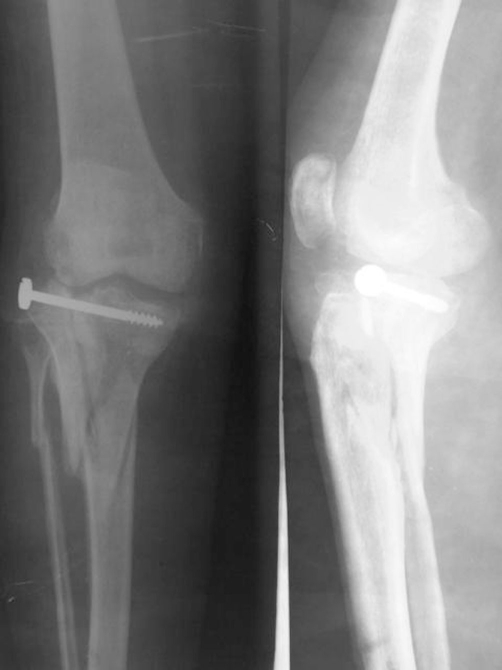
The reconstituted tibial plateau.

A Schanz pin was placed into the tongue shaped calcaneal fragment, starting posteriorly. This pin was attached to the second ring by means of a plate and two threaded rods. By gradual distraction and lateral pressure under image intensifier control, the Bohler angle was restored. The Schanz pin was kept in this position in the distraction mode. The patient was allowed range of motion exercises of the knee from the first post operative day. At 10 weeks the fixator was removed and patient allowed partial weight bearing crutch walking. 12 weeks post fixation full weight bearing was allowed (Figures 6 & 7). At final follow up of 40 weeks, the patient had a range of motion from 10 degrees to 125 degrees. The ankle range of motion at was 15 degrees dorsiflexion to 30 degrees of plantar flexion. The patient is able to ambulate without pain, and is able to do light activity. However, heavy activity causes mild discomfort around the knee.

## Discussion

High energy fractures of the tibial plateau and the calcaneum challenge the orthopaedic surgeon due to the difficulties in restoring the complex bony architecture and the tenuous nature of the soft tissues.

Cotton and Wilson in 1916 wrote that the man who breaks his heel bone is done [3]. The management of displaced, intra articular calcaneal fractures continues to elicit debate. Open reduction has not consistently improved outcome when compared with non operative treatment and in some cases has resulted in worse outcome [4]. The consensus however is that the treatment should attempt to improve the natural history of the injury.

Closed manipulation of the calcaneum has been recommended [5]. Paley et al. described minimally open procedures using the circular ring fixator for fixation [6].Closed manipulation primarily can be used for tongue type fractures and can restore the Bohler's angle. Tongue type fractures have a better outcome than joint depression injuries and Bohler's angle restoration often correlates with a better functional outcome [7,8]. The clinical importance of posterior facet congruity is supported by biomechanical data [9].

Low energy tibial plateau fractures have excellent clinical outcomes with few complications with contemporary internal fixation, however in high energy fractures; these methods produce severe complications [2]. The methodology of hybrid fixation has gained in popularity in cases with significant soft tissue injury, particular fragments and diaphysiometaphyseal extension.

The principles of bony reconstruction, particularly in weight bearing joints, include anatomic reduction and rigid internal fixation of intra-articular fractures and accurate restoration of coronal, sagittal and transverse mechanical axes. [1]. High energy fractures, including the Schatzker type VI, are best reduced by application of the principal of ligamentotaxis. The less invasive skeletal stabilization provides pre contoured plates with a locking screw plate interface resulting in a fixed angle implant. Sub muscular placement with percutaneous screw application is the advantage of this system. However the requirement of exposure for articular reduction and difficulty in reducing the posteromedial fragment are the difficulties encountered with this system.

The advantages of using hybrid fixation with a circular small wire external fixator are numerous, with minimal additional devitalisation, capture of very small metaphyseal and sub chondral fragments with lag effect [6,10]. However anatomic reduction is not always obtained due to the inability of ligamentotaxis to reduce all fragments. In our patient, osteoporosis made absolute anatomic reduction difficult to achieve. However the fracture was reduced to within the acceptable limits of 5 mm. We applied only two rings to the tibia with the two Schanz pins in the diaphysis producing significant stability in allowing proximal and distal adjustment.

In our case the fixator was able to manage both fractures simultaneously. The fixator permitted early range of motion, adjustability and observation. The Ilizarov fixator can be an important methodology in the management of such coexisting injuries.

## Conclusion

The circular wire fixator provides a viable method to manage such fractures especially if they are co existent. This is especially true in case the soft tissue envelop is compromised.

## Abbreviations

None.
